# Counterintuitive Range Shifts May Be Explained by Climate Induced Changes in Biotic Interactions

**DOI:** 10.1111/gcb.70332

**Published:** 2025-07-08

**Authors:** Inna Osmolovsky, Zoe A. Xirocostas, Giancarlo M. Chiarenza, Angela T. Moles

**Affiliations:** ^1^ Evolution & Ecology Research Centre, School of Biological, Earth and Environmental Sciences UNSW Sydney Sydney New South Wales Australia; ^2^ School of Life Sciences University of Technology Sydney Sydney New South Wales Australia; ^3^ ARC Training Centre for Healing Country, Department of Molecular and Life Sciences Curtin University Perth Western Australia Australia

**Keywords:** biotic interactions, climate change, downhill, equatorward, interaction opportunists hypothesis, range shifts

## Abstract

Many organisms are expected to shift their ranges uphill, toward the poles or to deeper waters in response to climate change. However, over a third of species exhibit counterintuitive range shifts—toward the equator, downhill or to shallower waters. Despite the prevalence and potential importance of counterintuitive shifts, they are seldom predicted by the species distribution models on which conservation decisions often rely, and we have remarkably few hypotheses as to why species might exhibit counterintuitive shifts. To address this, we propose the ‘Interaction Opportunists Hypothesis’, which formalises the idea that counterintuitive shifts could arise from climate change induced changes in biotic interactions at the warm edge of species' distributions. Reductions in antagonistic interactions, increases in positive interactions or changes in the type or outcome of biotic interactions could make previously unsuitable habitats viable parts of a species' range. Biotic interactions may additionally drive lags in range shifts and the persistence of some species in current habitats despite the changing climate. Understanding the role of biotic interactions is thus crucial for improving forecasting of the rate, direction and vulnerability of range shifting species, aiding conservation and climate mitigation efforts. Our hypothesis provides a generalisable framework to explain counterintuitive shifts across diverse systems and contexts.

## Graphical abstract

Climate change is expected to drive species uphill, poleward or into deeper waters. Yet many are shifting in toward the equator, downhill or to shallower waters, which is opposite to the expected direction under climate change. We propose that these unexpected movements may result from climate‐driven changes in interactions of the shifting species with other organisms, which make otherwise unsuitable habitats more favourable.

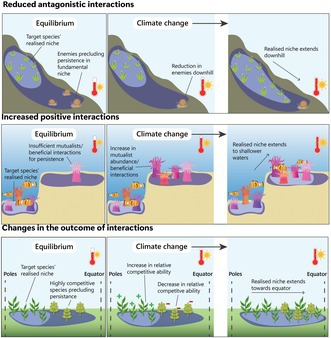

## 1 Introduction

Climate change is predicted to drive species uphill, toward the poles or to deeper waters (Kullman 2002; Lenoir and Svenning 2015; Peñuelas and Boada 2003; Walther et al. 2005). However, data compilation studies have consistently reported that 20%–37% of species are shifting downhill, toward the equator or to shallower waters (Lenoir et al. 2010; Osmolovsky et al. n.d; Parmesan and Yohe 2003; Rubenstein et al. 2023). That is, a substantial proportion of species are shifting in the opposite direction to what we expect under climate change. While there have been some attempts to explain counterintuitive shifts (Crimmins et al. 2011; Kuhn et al. 2016; Lawlor et al. 2024; Lenoir et al. 2010; Madsen‐Hepp et al. 2023; Neate‐Clegg et al. 2021; Pinsky et al. 2013; Zu et al. 2022), we have little generalisable theory, and counterintuitive range shifts are often overlooked or disregarded (Bates et al. 2015; Beissinger and Riddell 2021; Brown et al. 2016). This is a problem, as the species distribution models and theory on which many conservation decisions are predicated rarely predict downhill, equatorward or shifts to deeper waters (Archis et al. 2018; Biella et al. 2017; Lee‐Yaw et al. 2022; but see Tagliari et al. 2021; Zhang et al. 2019).

Species Distribution Models and most current theory are focused on climate as the main driver of range shifts (Araújo and Peterson 2012; Oldfather et al. 2020; Rapacciuolo et al. 2014; Thuiller et al. 2005; Urban 2015). While species' distributions are shaped by abiotic factors (e.g., temperature, precipitation and water regimes, soil fertility), biotic interactions such as facilitation, pollination, predation, foraging, competition and/or interactions with pathogens or parasites can also influence species ranges (Alexander et al. 2015; Anthelme et al. 2014; Bretagnolle and Gillis 2010; Brown and Vellend 2014; Bullock et al. 2000; Fine et al. 2004; Fowler et al. 2023; Hargreaves and Eckert 2014; HilleRisLambers et al. 2013; Louthan et al. 2015; Lyu and Alexander 2022; Paquette and Hargreaves 2021; Sexton et al. 2009; Tylianakis et al. 2008; Zhuo et al. 2025). Previous research has considered several ways that biotic interactions can impact how species shift their ranges in response to climate change (Alexander et al. 2015; Lavergne et al. 2010; Lenoir et al. 2010; Tomiolo and Ward 2018). Biotic interactions can contribute to lags in response to climate change (Hegland et al. 2009; Hellmann et al. 2012; Lavergne et al. 2010), range contractions or extinctions (Le Roux et al. 2012; Urban et al. 2013). Biotic interactions may promote faster range expansions (Cleavitt et al. 2022; McCarthy‐Neumann and Ibáñez 2012; Urli et al. 2016). The incorporation of the distribution of an interacting species often improves models' predictive accuracy (Meineri et al. 2012; Pellissier et al. 2010; Preston et al. 2008; Urban et al. 2013); however, predictive accuracy improved even when including the distribution of other, non‐interacting species (Giannini et al. 2013). Thus, we still have much to learn about how biotic interactions drive range shifts (Elith et al. 2006; Elith and Leathwick 2009).

## 2 The Interaction Opportunists Hypothesis

**FIGURE 1:**
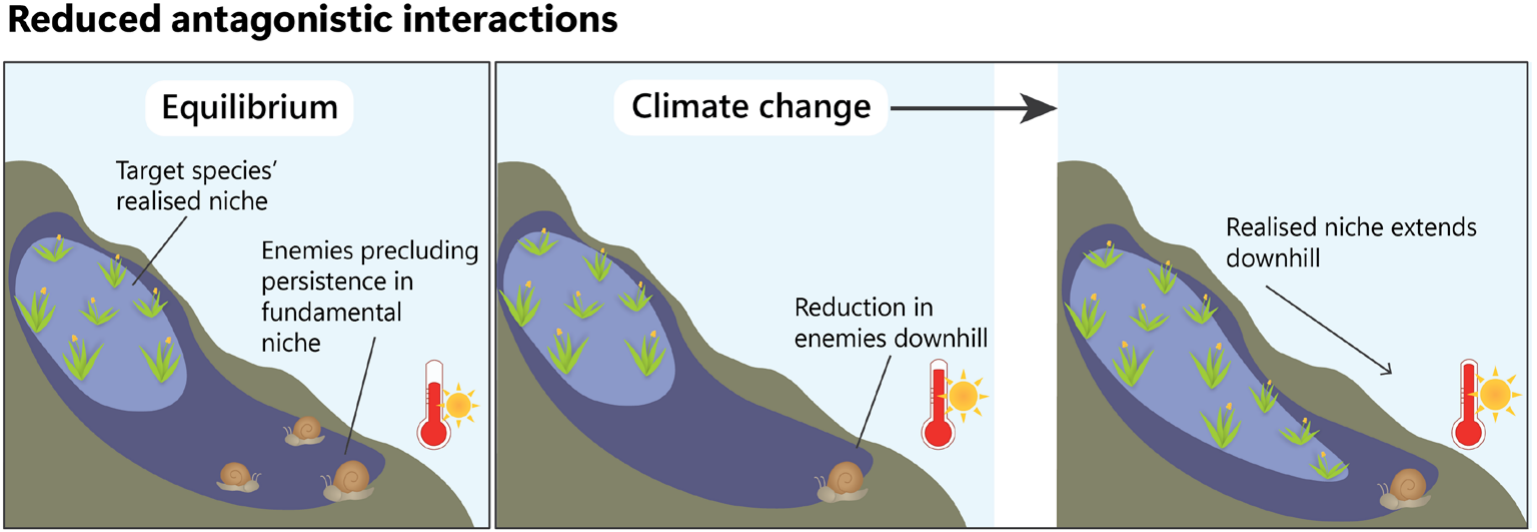
Conceptual illustration of how a climate change‐driven reduction in antagonistic biotic interactions can drive counterintuitive range shifts.

**FIGURE 2:**
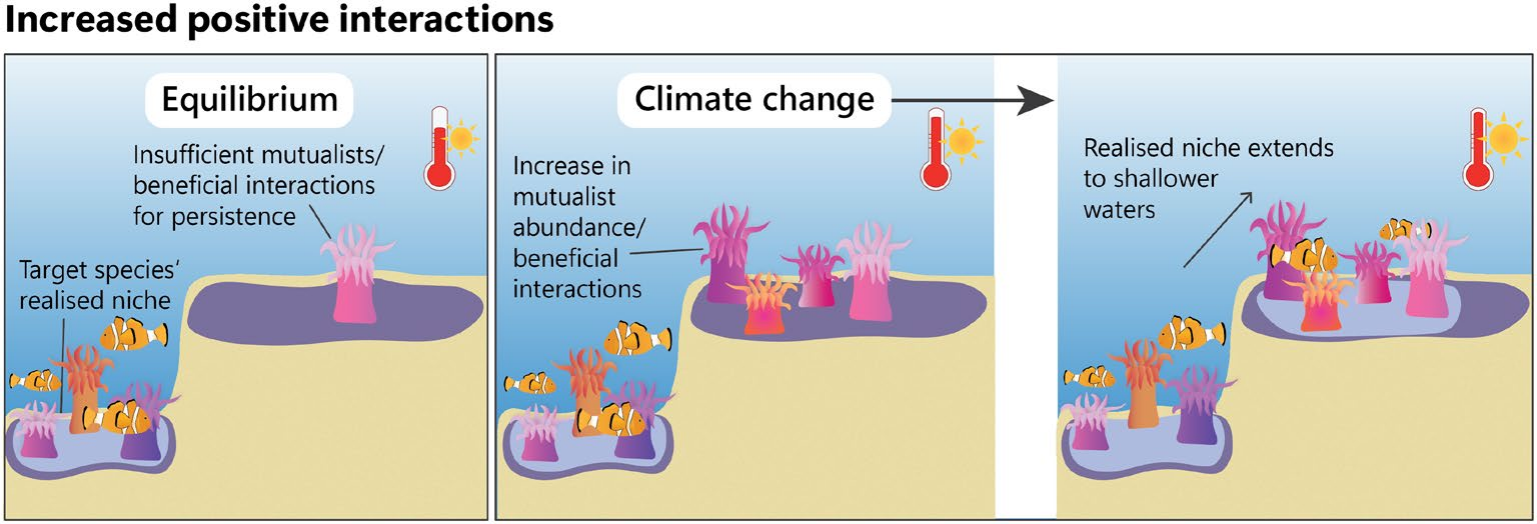
Conceptual illustration of how a climate change‐driven increase in positive interactions can drive counterintuitive range shifts.

**FIGURE 3:**
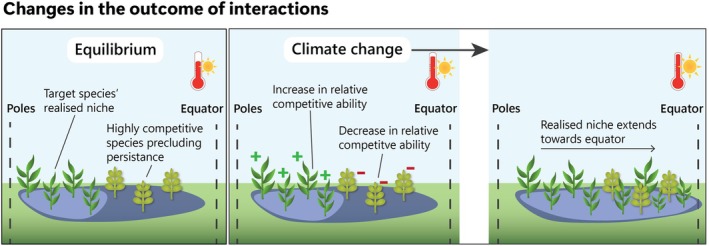
A conceptual illustration on how climate change‐driven change in the outcome of a biotic interaction can drive counterintuitive shifts.

We formalise the idea that climate change can drive counterintuitive range shifts by altering biotic interactions, subsequently inducing favourable conditions beyond the species' warm range edge (Figures 1, 2, 3). There is strong evidence that climate change is altering the number, strength and nature of biotic interactions (Bale et al. 2002; Fontúrbel et al. 2021; Wisz et al. 2013). Climate change‐induced changes in biotic interactions might sometimes allow species to expand their ranges in counterintuitive directions in three main ways: (1) reduced antagonistic interactions, such as herbivory, pathogen attack, parasitism, competition or predation (Figure 1); (2) increased positive interactions, such as increased availability or effectiveness of mutualists or increased abundance or quality of prey (Figure 2); (3) changes in the outcome of interactions, such as changes in species' relative competitive ability or changes in the cost/benefit ratio in mutualisms (Figure 3). We discuss these possibilities below.

### 2.1 Reduced Antagonistic Interactions

Under the stress trade‐off hypothesis, the ‘warm’ edge of a species' distribution is more strongly limited by biotic interactions such as competition and predation, while the ‘cold’ edge is predominantly restricted by climate (Choler et al. 2001; Hampe and Petit 2005; Hargreaves et al. 2014; HilleRisLambers et al. 2013; Klanderud et al. 2021; Paquette and Hargreaves 2021; but see Rinas and Vellend 2024). Biotic interactions are proposed to be stronger at lower elevations, lower latitudes and in shallower waters (Hargreaves et al. 2014; HilleRisLambers et al. 2013; Paquette and Hargreaves 2021). If the antagonistic interactions that limited species' distributions are reduced because of climate change, species might be able to expand their range downhill, equatorward or to shallow waters. It has previously been suggested that changes in interspecific competition could facilitate counterintuitive shifts (Foster and D'Amato 2015; Lawlor et al. 2024; Lenoir et al. 2010). Here, we extend this idea to a range of other antagonistic interactions. Pathogens, predators and herbivores may limit the distribution of their victims by reducing their fitness below the establishment and dispersal thresholds, but not depleting the populations completely (Holt and Barfield 2009). For example, herbivores and predators may target early or reproductive life stages of their target prey species, limiting further dispersal and establishment (Brown and Vellend 2014; Holt and Barfield 2009). Similarly, predators may reduce prey fitness by limiting access to resources such as food (Beck et al. 2016; Holt and Barfield 2009). Empirical evidence confirms that plant ranges can be limited by herbivory (Benning et al. 2019; Benning and Moeller 2021; Brown and Vellend 2014; Fine et al. 2004; Ni and Vellend 2024), predators can limit the distribution of their prey (Durant et al. 2023; Harley 2011) and pathogens and parasites can limit the distribution of various host taxa (Anderson 1972; van der Putten et al. 2007). A reduction in antagonistic interactions might arise from different rates and magnitudes of responses to climate change between different species. For example, the antagonists may shift their ranges uphill, poleward or to deeper waters, vacating previously unavailable parts of the fundamental niche (Lawlor et al. 2024). Alternatively, alleviation of biotic constraints may arise from local extinction of the antagonists or from changes in phenology that would drive temporal mismatch between previously interacting species (Bretagnolle and Gillis 2010).

### 2.2 Increased Positive Interactions

Although the role of mutualistic interactions in limiting species' distributions was weakly supported by data in two systematic reviews (Abeli et al. 2014; Dawson‐Glass and Hargreaves 2022), a low abundance of mutualists can limit species' distributions (Burke et al. 2019; Duffy and Johnson 2017; Fowler et al. 2023; Moeller et al. 2012). Climate change could potentially lead to an increase in the frequency or efficacy of positive interactions. Increased positive interactions may help species access parts of their fundamental niche that were previously unsuitable (Fowler et al. 2023; HilleRisLambers et al. 2013; Parmesan and Yohe 2003), subsequently promoting counterintuitive range expansions (Figure 2). For example, an increased abundance of pollinators, food sources (palatable plants or prey), seed dispersers, mycorrhizae and facilitators might mitigate unsuitable climatic conditions and increase fitness (Afkhami et al. 2014; Anthelme et al. 2014; Bretagnolle and Gillis 2010; Connor 1995; Fowler et al. 2023; Pinsky et al. 2020; Tourville et al. 2024) and/or increase dispersal and establishment potential (Chuang and Peterson 2016; Fowler et al. 2023; Hampe 2011). Increased positive interactions could arise from the range expansions or changes in abundance of native species or from the arrival of novel, non‐native species (Gérard et al. 2015; Gilbert and Parker 2010).

### 2.3 Changes in the Outcome of Interactions

The final way that climate change induced changes in biotic interactions could yield counterintuitive shifts in species distributions is by changing the outcome of biotic interactions. According to the stress‐gradient hypothesis, an increase in abiotic stress, such as that imposed by climate change, can turn antagonistic interactions into positive interactions (Bertness and Callaway 1994; Maestre et al. 2009). For example, the pathogenic barley yellow dwarf virus benefits its host when under acute water stress, with inoculated wheat growing larger and producing more and heavier seeds (Davis et al. 2015). Antagonistic interactions frequently switch to positive interactions with increased environmental stress (Adams et al. 2022; Choler et al. 2001; He et al. 2013; Klanderud et al. 2021; Lortie and Callaway 2006; Olsen et al. 2016; but see Maestre et al. 2005), although environmental stress can cause organisms to switch from positive to harmful interactions or for mutualists to leave their hosts entirely (Toby Kiers et al. 2010). Climate change may contribute to the rewiring of ecological networks, such as changes in the hierarchy of competitive strength among species with different species becoming competitively dominant under certain environmental conditions (Alexander et al. 2015). Despite the negative effects on some species, community‐wide rewiring could permeate across ecological communities and networks, contributing indirectly to higher fitness and potential range expansions in some species.

### 2.4 Evidence in Support of the Interaction Opportunists Hypothesis

Empirical evidence for the importance of changes in biotic interactions in promoting counterintuitive shifts is scarce. We found only two studies either consistent with or counter to the Interaction Opportunists Hypothesis, and both involve changes in biotic interactions in response to altered anthropogenic pressures rather than climate change. One study suggested that native plants in the Nepalese Himalayas shifted downhill following a reduction in grazing (Bhatta et al. 2018). The other study reported that *Tamiasciurus hudsonicus* , a squirrel native to North America, is shifting downhill following the recovery of a red‐spruce forest (Morelli et al. 2025).

## 3 Discussion

Previous explanations of counterintuitive shifts have tended to be situation specific. For example, local topography drove some plant species to shift downhill as they were shifting northward in France (Kuhn et al. 2016). An increase in precipitation at lower elevations drove species to shift downhill in California, China and Kenya (Crimmins et al. 2011; Fu et al. 2024; Zu et al. 2022). Marine species in North America tracked changes in local rather than region‐wide climates, exhibiting counteractive shifts (Pinsky et al. 2013). Similarly, traits showed correlation with downhill range shifts in local studies (Madsen‐Hepp et al. 2023; Neate‐Clegg et al. 2021), but not in cross‐continental analyses (Angert et al. 2011; MacLean and Beissinger 2017; Osmolovsky et al. In Prep). Finally, antagonistic biotic interactions, such as competition and predation, have been proposed to drive counterintuitive shifts (Lawlor et al. 2024; Lenoir et al. 2010), often in combination with changes in human‐mediated disturbance (Bhatta et al. 2018; Foster and D'Amato 2015; Lenoir et al. 2010). While all of these factors could be important in some situations, arriving at idiosyncratic explanations for each counterintuitive shift is unsatisfying, particularly considering the high prevalence of counterintuitive shifts worldwide (Lenoir et al. 2010; Osmolovsky et al. In Prep; Parmesan and Yohe 2003). One appealing aspect of the Interaction Opportunists Hypothesis is that it is applicable across different species, ecological communities, ecosystems and biomes and thus might provide a coherent explanation for many counterintuitive shifts worldwide (particularly in situations where species' fundamental niche is much broader than their realised niche, and where climate change affects interacting partners more than the focal species).

The changes in biotic interactions contributing to counterintuitive shifts in the Interaction Opportunists Hypothesis have parallels to processes contributing to range expansion in introduced species, such as enemy release (Keane and Crawley 2002). However, there is an important difference between these mechanisms. Where introduced species move to a new environment and simultaneously undergo changes in biotic interactions (Keane and Crawley 2002), under the Interaction Opportunists Hypothesis, species would first experience changes in ecological dynamics at their warm edge, which will subsequently drive the counterintuitive range shift (Lenoir et al. 2010).

The Interaction Opportunists Hypothesis can be tested using standard approaches to quantify species' interactions, including surveys of the dynamics and patterns of biotic interactions across the range of actively shifting and non‐shifting species (Hargreaves et al. 2015; Paquette and Hargreaves 2021; van Grunsven et al. 2010), transplant experiments (Bektaş et al. 2024; Brown and Vellend 2014; Hargreaves et al. 2014; Rivest and Vellend 2018) and computer‐based modelling (Araújo and Luoto 2007; Burke et al. 2019; Duffy and Johnson 2017; Sanczuk et al. 2024). The importance of climate change‐induced alterations of biotic interactions in driving counterintuitive range shifts could also be gauged by comparing historic records of biotic interactions to current dynamics (e.g., resurveying sites in which biotic interactions were quantified in the past to estimate changes in interactions) at the warm range edges of species that are shifting in counterintuitive vs. predicted directions.

One interesting question is whether counterintuitive shifts are beneficial for the long‐term prospects of the species, or if shifts to warmer areas simply delay species declines (i.e., do counterintuitive shifts contribute to extinction debt?; Stork 2010; Tilman et al. 1994). Extinction debt may be particularly acute in counterintuitively shifting species because they are dispersing toward their potential niche limitations, whereas species shifting uphill, poleward or to deeper waters may better track their climatic requirements (Rumpf et al. 2019). The climate is likely to continue changing and as such may eventually make the species' entire range climatically unsuitable, leading to an abrupt extinction of counterintuitively shifting species, despite the recently improved biotic conditions. On the other hand, counterintuitively shifting species may still benefit from a temporary range expansion. A larger range that includes different habitats and more individuals of a species could contribute to increased adaptive and evolutionary potential in the face of climate change, thus improving species' chances to cope with more adverse climatic conditions (Jump and Peñuelas 2005). Gene flow from the warm range edge to core and cold edge individuals may increase a species' overall ability to cope with warmer climates, as warm edge individuals may be particularly adapted to withstand higher temperatures (Kremer et al. 2012). Further, species with large distributions and larger populations are expected to face lower extinction risk (Soulé 1987; Staude et al. 2019).

The Interaction Opportunists Hypothesis focuses on range expansion at the warm range edge and does not make predictions about potential changes in distribution at the core and cold range edge of species distribution. While species may experience uniform changes in their distribution across their ranges (Auld et al. 2022; Lenoir et al. 2008), more studies propose that range shifts may vary across different parts of species' distributions (Comte et al. 2014; Fréjaville et al. 2020; Lenoir et al. 2020). Biotic interactions may play a role in how species shift their ranges in all parts of their distribution, affecting the size of species' ranges and their resilience in the face of climate change.

Reduced antagonistic interactions or increases in positive biotic interactions could contribute to range shifts in both expected and counterintuitive directions (Lyu and Alexander 2022). For example, empirical studies show that species shifting their ranges uphill or poleward are losing their enemies (Hellmann et al. 2012; Morriën and van der Putten 2013; van Grunsven et al. 2010). The reduced antagonistic pressure could lead to further or faster range expansions (Cleavitt et al. 2022; McCarthy‐Neumann and Ibáñez 2012; Urli et al. 2016). Similar pathways could emerge through increased positive interactions. Thus, in addition to explaining counterintuitive shifts, accounting for changed biotic interactions could increase the accuracy of model predictions for species that are shifting uphill, to deeper waters or to higher latitudes.

A mismatch between the response rates of interacting species could contribute to lags in the rate at which species are shifting their ranges (Hegland et al. 2009; Hellmann et al. 2012; Lavergne et al. 2010). If this is the case, incorporating biotic interactions in our models will help us to understand why some species are not shifting their distributions or are shifting more slowly than expected. This is an important area for improved model fits, as lags are prevalent across many taxa and biomes (Bertrand et al. 2011; Bertrand et al. 2016; Lenoir et al. 2020), with one study proposing that plant species in the US shift at 52% of the rate required to track the rate of climate change (Ash et al. 2017). Although several studies explore the role of biotic interactions in limiting species distributions and in shaping future shifts in distributions (Bektaş et al. 2024; Brown and Vellend 2014; Dawson‐Glass and Hargreaves 2022; Hargreaves et al. 2015; Moeller et al. 2012; Morriën and van der Putten 2013; van Grunsven et al. 2010), the evidence of biotic interactions and mismatches with symbionts is still mainly theorised. It is thus crucial to understand the role of biotic interactions in shaping not only the direction but also the rate at which species are shifting in response to climate change.

Climate change is increasing the stochasticity of our ecosystems (Rollinson et al. 2021), posing novel challenges to our ability to anticipate species responses and inform conservation practices. Traditionally, predictions of species' responses to climate change relied solely on climatic variables and the thermal tolerance limits of species (Araújo and Peterson 2012; Elith and Leathwick 2009; Jeschke and Strayer 2008). However, mounting evidence suggests that species' responses are shaped by more than just climate (Gibson‐Reinemer and Rahel 2015; Lavergne et al. 2010; Tomiolo and Ward 2018; van der Putten 2012). It is our hope that increasing the focus on changing biotic interactions improves researchers' ability to accurately predict species' responses to climate change, thus enabling more effective conservation decisions, management strategies and ecological outcomes.

## Author Contributions

**Inna Osmolovsky:** conceptualization, project administration, visualization, writing – original draft, writing – review and editing. **Zoe A. Xirocostas:** visualization, writing – original draft, writing – review and editing. **Giancarlo M. Chiarenza:** writing – original draft, writing – review and editing. **Angela T. Moles:** conceptualization, visualization, writing – original draft, writing – review and editing.

## Conflicts of Interest

The authors declare no conflicts of interest.

## Data Availability

The authors have nothing to report.
